# Modelling Noninvasively Measured Cerebral Signals during a Hypoxemia Challenge: Steps towards Individualised Modelling

**DOI:** 10.1371/journal.pone.0038297

**Published:** 2012-06-05

**Authors:** Beth Jelfs, Murad Banaji, Ilias Tachtsidis, Chris E. Cooper, Clare E. Elwell

**Affiliations:** 1 Department of Medical Physics and Bioengineering, University College London, London, United Kingdom; 2 Department of Mathematics, University of Portsmouth, Portsmouth, United Kingdom; 3 School of Biological Sciences, University of Essex, Colchester, United Kingdom; Glasgow University, United Kingdom

## Abstract

Noninvasive approaches to measuring cerebral circulation and metabolism are crucial to furthering our understanding of brain function. These approaches also have considerable potential for clinical use “at the bedside”. However, a highly nontrivial task and precondition if such methods are to be used routinely is the robust physiological interpretation of the data. In this paper, we explore the ability of a previously developed model of brain circulation and metabolism to explain and predict quantitatively the responses of physiological signals. The five signals all noninvasively-measured during hypoxemia in healthy volunteers include four signals measured using near-infrared spectroscopy along with middle cerebral artery blood flow measured using transcranial Doppler flowmetry. We show that optimising the model using partial data from an individual can increase its predictive power thus aiding the interpretation of NIRS signals in individuals. At the same time such optimisation can also help refine model parametrisation and provide confidence intervals on model parameters. Discrepancies between model and data which persist despite model optimisation are used to flag up important questions concerning the underlying physiology, and the reliability and physiological meaning of the signals.

## Introduction

Improvements in noninvasive approaches for measuring cerebral circulation and metabolism have the potential to significantly increase our understanding of the healthy and injured brain. Of current interest is the use of near-infrared spectroscopy (NIRS) to monitor simultaneously brain oxygenation, haemodynamics and metabolism [1]–[4], particularly as part of a multimodal monitoring strategy in neurointensive care [5]. In order to guide patient management, the robust extraction of clinically relevant information from these systems is key. However, interpretation of NIRS and other measured signals requires both considerable knowledge of the underlying physiology, and an understanding of the physics of the measurement process itself. In situations such as these where understanding *in vivo* physiology requires measurement whose interpretation is itself dependent on an understanding of the physiology we naturally require methodologies where modelling, both of physiology and of the measurement process, play a key role.

In this paper, the predictions of a previously developed model of brain circulation and metabolism [6], termed BrainSignals, are compared with experimentally measured data from ten healthy volunteers each undergoing a series of three hypoxemic challenges as described in [7]. BrainSignals itself was constructed and parametrised primarily using published data, much of it from *in vitro* experiments, and hence of a very different kind to the *in vivo* measurements presented here. Thus one goal is to evaluate the performance of BrainSignals in an *in vivo* context where there is considerable physiological and measurement noise, and repeatable, controlled experiments are impossible. Preliminary studies [8] suggested that in this context qualitative trends in certain signals could be predicted with some consistency by the model. Here, the aim is to carry out a more complete, quantitative, comparison of data and model prediction, using both default and optimised model parameters. This work should be seen in the broad context of an iterative process of model development and comparison to data gradually leading to convergence between model predictions and measurements.

A key aspect if a model is ever to be used at the bedside in a clinical context is that it must be able to inform not only on averaged behaviour, but also on the behaviour of individuals who will display a wide range of natural physiological and pathophysiological variation. The level of success of subject-specific optimisations of physiology-based models, particularly those of some complexity, is an important topic going beyond the specifics of cerebral circulation and metabolism described here. Making a model subject-specific involves reparametrisation in such a way as to maximise its ability to describe the physiology of a particular individual. In this context, success of the model is characterised via its ability to reproduce data for an individual subject, given available prior information/data on the subject. Here, the prior information takes the form of a part of the experimental data-set with the goal being to optimise the model using this data in such a way as to improve the predictive capabilities of the model for the rest of the data set. Hence the work presented here can be seen as a step towards a more ambitious, longer-term aim of “subject-specific” modelling. In particular our goals are:

1. To quantify the predictive capabilities of the model for each of the different signals.
2. To find out to what extent model reparametrisation can improve model predictions.
3. To determine the robustness with which physiologically important model parameters can be determined for an individual from data.
4. To identify systematic discrepancies between model predictions and data and to speculate about the origins and resolutions of these inconsistencies.
5. To generate testable hypotheses about how physiologically important, but hard to measure, quantities might behave during hypoxia.

However, it is important to clarify at the outset that we are neither attempting to “validate” the model against the measurements, nor to “validate” the measurement technologies with the aid of the model.

## Materials and Methods

### Description of the Experiment

This study was approved by the Joint Research Ethics Committee of the National Hospital for Neurology and Neurosurgery and the Institute of Neurology. The data analysed here is from a study of 10 healthy subjects (7 male, 3 female, median age 32 years, range 30–39) for which all subjects gave written informed consent. The details of the experimental protocols and measurement methodologies are provided in previous publications [7], [9]. In brief, inspired oxygen concentration 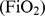 was measured using an inline gas analyser, and a pulse oximeter probe measured arterial oxygen saturation 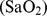. The study commenced with five minutes monitoring at normoxia. Then nitrogen was added to the inspired gases with the aim of inducing a gradual fall in 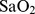 to approximately 80%; immediately after this was achieved, 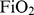 was returned to normal for five minutes. This cycle was repeated three times. Throughout the study, end tidal carbon dioxide tension 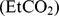 and breathing rate were measured continuously and fed back to subjects in order to adjust their minute ventilation to maintain normocapnea. Heart rate and mean arterial blood pressure (ABP) were also measured continuously.

A combination of two continuous wave near-infrared spectrometers, an in-house developed broadband spectrometer (BBS) previously described by Tisdall et al. [7], and the commercially available NIRO 300 (Hamamatsu Photonics KK), were used in conjunction with transcranial Doppler (TCD) ultrasonography to monitor brain tissue haemodynamics, oxygenation and metabolism [10]. Further details of the NIRS signals are given in the following section. Mean velocity of blood (vMCA) was measured in the basal right middle cerebral artery.

For the purposes of input to the model, and comparison with model output, measured signals were preprocessed as follows: all signals were visually inspected for well-characterised artifacts, which, if present were manually removed; all signals were low pass filtered to remove high frequency instrumentation and physiological noise using a 5th order Butterworth filter with a cut-off frequency of 0.1 Hz; all signals were resampled to 1 Hz; and linear detrending was carried out on the differential spectroscopy signals.

From here on, the subjects will be referred to as Subject 1 to Subject 10. The full set of three hypoxemic challenges carried out on a subject will be termed an **experiment**, and a single hypoxemic challenge will be referred to as a **challenge**. Thus 10 experiments each involving 3 challenges were carried out, giving a total of 30 challenges. For the purposes of analysis, the division of each experiment into three challenges was carried out manually by choosing time-points in the middle of the periods of normoxia between hypoxemic challenges, and cutting all data-sets at these points.

### Near-Infrared Spectroscopy

Four out of the five signals considered here are measured using NIRS. Changes in tissue oxy- and deoxy-haemoglobin concentrations, termed 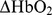 and 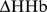 respectively, can be measured using differential spectroscopy systems [11]–[13]. In the analysis here, rather than directly using 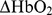 and 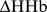, two derived quantities, the total haemoglobin concentration change 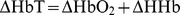, and changes in the difference 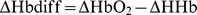 are used. The third signal under consideration is the absolute tissue oxygen saturation (TOS), which provides a percentage measure of mean oxygen saturation across all vascular compartments in the region of tissue queried. TOS has been used extensively as a marker of tissue oxygenation in a range of applications [14]–[17] but its relationship to underlying physiology is still under investigation [18]–[20]. Finally, in addition to the haemoglobin chromophores, the 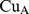 centre in cytochrome *c* oxidase (CCO) is a significant NIR absorber. Changes in oxidation of this centre give rise to the fourth NIRS signal considered here, referred to as 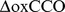. Interpretation of the physiological meaning of 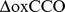 is, however, nontrivial. This signal has been extensively investigated as a marker of cellular oxygen metabolism [21]–[24], and a number of clinical studies have been performed to elucidate its role as a measure of cerebral well being [5], [25], [26]. Theoretical, qualitative analysis of how it responds to changes in oxygenation, substrate supply, and metabolic demand were carried out in [27], with more quantitative discussion in [6].

### The BrainSignals Model

The BrainSignals model is described in [6], and available for download at [28]. This model is a simplification of the large scale BRAINCIRC model in [29] and was constructed to aid prediction of a number of measurable signals (including those described above), thus allowing model performance to be better evaluated against *in vivo* data and maximise the clinical relevance of the previous modelling work. At the same time, model complexity was minimised by removing or simplifying components of the physiology regarded as nonessential to the basic observed behaviours. This simplification resulted in a model consisting of two components: a submodel of the cerebral circulation, which is known to respond in complicated ways to a variety of stimuli [30], and a submodel of mitochondrial metabolism related to those presented in [31], [32]. The two components are linked via the processes of oxygen transport and consumption. Where possible, model parameters were chosen to be consistent with thermodynamic principles and *in vitro* data. Necessarily, a number of parameters which were either expected to have wide physiological variation between individuals or can be hard to measure were given “typical” values, in the anticipation that they could be set via more extensive comparison with *in vivo* data, as carried out here.

#### Inputs and Outputs of the Model

**Figure 1:**
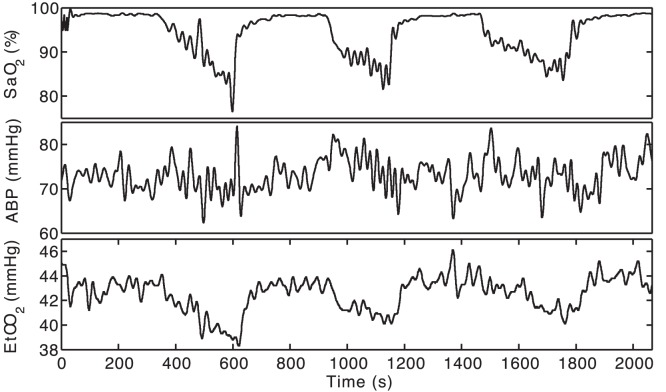
Typical input traces. 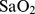
 (%, top panel), mean ABP (mmHg, middle panel) and 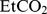 (mmHg, bottom panel) for a typical subject (Subject 6) following the resampling and filtering described in the text. Each experiment lasted 30–40 minutes in total.

**Figure 2:**
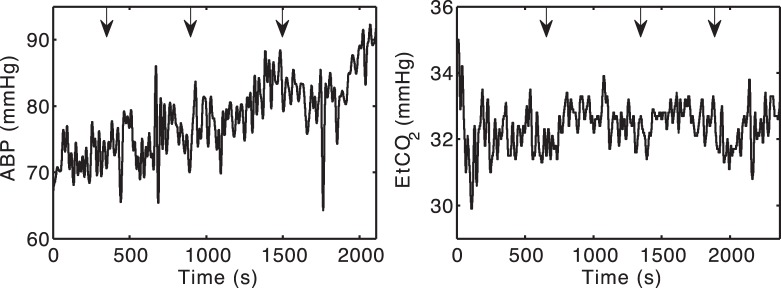
Examples of behaviour of the input signals. In both figures, arrows indicate the start of each hypoxemic challenge. *Left.* The mean arterial blood pressure trace for Subject 2 showing a marked increase during the experiment. *Right.* The end tidal 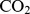 tension for Subject 5 was maintained relatively constant across the hypoxemic challenges.

Inputs to the BrainSignals model were three measured systemic signals: mean ABP, 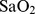 and 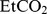 (which was assumed to be equal to arterial partial pressure of 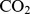, a model input parameter). Fig. 1 shows typical input data for one subject (Subject 6). Note that the experimental protocol meant that each hypoxemic episode was neither necessarily of the same magnitude nor of the same duration. Thus extrapolating from one hypoxemic challenge to the next becomes nontrivial. Note further that a single challenge cannot simply be treated as a drop in oxygen, as a number of other systemic effects may be simultaneously occurring. These included significant changes in mean blood pressure, and in some cases significant changes in 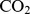 levels. These collateral effects were extremely variable across the subject range. For example Subject 2 showed a rise in blood pressure during the experiment (Fig. 2, left), while Subject 5, unlike Subject 6 shown in Fig. 1, did not show significant 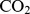 drops during the hypoxemic challenges (Fig. 2, right).

The four NIRS signals 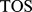, 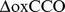, 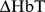 and 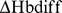 along with vMCA were used to provide a comparison between model and data. For the purposes of comparison between measured and modelled signals, vMCA was scaled by an arbitrary factor, which was chosen to equalise the average measured and modelled signals in each case. The three differential spectroscopy signals 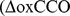, 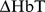 and 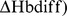 were subject to an arbitrary baseline shift again chosen to equalise the average signals. Measured and modelled TOS were compared without any scaling or baseline shift.

In addition to predicting the above five signals, the model produces a number of outputs which were not measured, and are in general difficult to measure in clinical contexts, including the levels of various chemical quantities in different compartments, and perhaps most importantly cerebral metabolic rate for oxygen 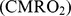. Model predictions of change in this quantity will be detailed in the results. The full range of model variables and outputs is described in [6].

#### Model optimisation

**Figure 3:**
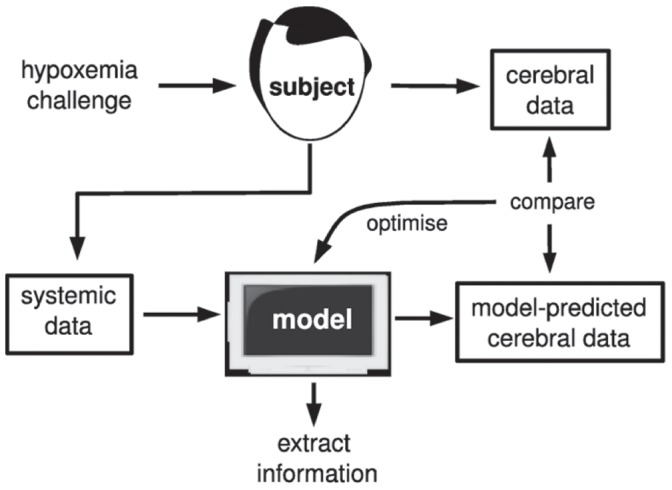
Schematic of the model optimisation methodology. Healthy subjects undergo a hypoxemic challenge, during which systemic and cerebral data is gathered noninvasively. The systemic data is fed into the physiological model, which then predicts expected values of the cerebral signals. The difference between measured and predicted values of these signals is used to construct an objective function, and minimisation of this function is used to reparametrise the model.

Carrying out model optimisation for a number of data sets, as done here, is an important means of identifying inadequacies of the model class, and problems with the measurement process. An outline of the optimisation process is presented in Fig. 3. Model optimisation broadly refers to attempting to minimise the distance between a set of model-predicted quantities and the corresponding measured quantities. The compatibility of a measured data set with a particular set of models (defined, for example, by free parameters in a model) is quantified by the minimal distance achieved through the optimisation process.

In this case to obtain the set of models for optimisation the parameters which most affected the model predictions of the five signals in question were determined through a dual approach. Discussion with clinical collaborators was used to establish which model quantities were potentially of most clinical use and also which quantities might be expected to show widest physiological variation. At the same time, a preliminary sensitivity analysis was carried out on a large range of model parameters in different scenarios. The seven optimisation parameters chosen through this process were:

1. The normal venous-arterial volume ratio, VARn (i.e., 1/AVRn in [6]). This can be expected both to vary from individual to individual and to be sensitive to the particular placing of the NIRS probes. Further, preliminary simulations showed that the haemoglobin-related NIRS quantities were sensitive to the values of this parameter.
2. Blood concentration of haemoglobin [Hb] (termed [Hbtot] in [6]), which can also be thought of as representing haematocrit. This is known to vary considerably between individuals. As with AVRn, haemoglobin-related NIRS quantities showed sensitivity to the values of this parameter.
3. A typical arteriolar radius 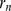. All haemodynamic model quantities were sensitive to values of this parameter, and for this reason it was included.
4. A dimensionless parameter 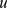, representing normal energy demand. All five outputs, including 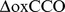, showed sensitivity to this parameter. However, preliminary simulations gave ambiguous results about whether it could be reliably determined from the data.

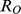
, the strength of cerebral blood flow (CBF) regulation in response to arterial 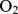 levels. Given the potential clinical importance of quantifying the regulatory response to changes in 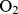 tension, we were keen to know whether this model parameter could be determined consistently for an individual from physiological data. Preliminary results were ambiguous, so it was included.

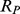
, the strength of CBF autoregulation in response to changes in blood pressure. Again, this is a quantity of potential clinical importance. Although the challenge did not involve any explicit manipulations of blood pressure, blood pressure often showed considerable spontaneous natural fluctuation during the challenge. Preliminary simulations suggested that this model parameter could be determined with some accuracy for an individual from physiological data.

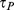
, the typical time-constant for the pressure autoregulation response. Preliminary investigation suggested that the flow responses (as recorded by vMCA) did not show the delays suggested by the literature on pressure autoregulation (e.g. [33], [34]). We wanted to find out if this was consistently the case.

Certain other parameters given initial consideration, for example, the typical time-constant for autoregulatory response to hypoxia, were found to be hard to determine from the data, and hence were excluded. The computational methodology used a simulated annealing approach using a simplex method, as described in [34]. This choice was considered appropriate given the large number of parameters being explored, the noisy nature of the data, and consequent uncertainty about the smoothness of the objective function. The initial temperature, the number of temperature drops, and the stopping criteria were determined heuristically. For the optimisations involving seven parameters and all five output signals, a maximum of 10,000 steps were allowed. When optimising for each signal individually a maximum of 1000 steps were allowed. All optimisations were run using the BRAINCIRC modelling environment [36].

Table 1 shows the model default values, and the lowest and highest values allowed by the optimisation process before a sliding penalty was applied, all other model parameters were fixed at default values. To avoid introducing undue bias into our results the penalty applied was only a weak one, the reasons for applying the penalty outside certain bounds were both to try to ensure that the parameters remained within physiological ranges, and also to ensure model stability. A graded penalty was applied to avoid discontinuities in the objective function thus stabilising the optimisation process.

**Table 1:** Optimisation parameters.

Parameter	Model default	Penalty below	Penalty above
VARn	3	1	5
**[Hb]** (mM)	9.1	5	12
*r_n_* (cm)	0.0187	0.012	0.022
u	1	0.1	4
*R_o_*	1.5	0.1	8
*R_p_*	4.0	0.5	10
*τ_p_* (s)	5.0	0.5	10
*V_ext_*	0	0.0	0.7

The model defaults of the free parameters and lowest and highest values permitted before the penalty was applied.

### Measuring the Success of the Optimisation Process

#### Comparing Model and Data

Given a particular signal, the mean distance between the measured and modelled values of this signal provides a quantification of the success of the model at reproducing that signal (mean distances as opposed to the RMS distances were chosen in order not to give undue weight to outliers). More precisely, given a signal 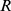, for subject 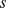, during challenge 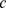, then 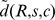, is the unweighted mean of the absolute difference between measured and modelled values of 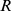 during the challenge (rescaled in the case of vMCA and with baseline shifted in the case of the differential spectroscopy signals).

It should be noted that the difficulties involved in robustly reproducing five signals simultaneously are considerably greater than in reproducing one or two signals. For a given set of signals, the weighted average of the distances across the signals provides a measure of the success of the model at reproducing the signals collectively. For two different signals 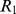 and 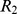 the distances 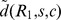 and 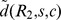 are not easily comparable as the signals are measured in different units and have different typical values, or typical changes during an experiment. In order to make the quantities comparable, we define weights 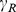, and weighted distances 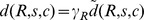. The total distance between model and data is then defined as a sum of the quantities 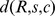.

For each of the thirty challenges individually, an optimisation was carried out to find the minimum achievable total distance between the model and the data. From these preliminary optimisations 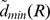, the smallest achievable values of 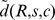 for each 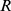 and across a range of subjects and challenges could be estimated. The weights 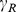 were then chosen in such a way that 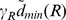 was similar for each of the five signals, for brevity this set of optimisations is termed **Fit 1**. This methodology can be seen both as normalising the signals, and also taking into account the relative ability of the model to fit the signals individually.

#### Prediction factors

An important question is whether the optimisation process improves the model’s ability to predict data unseen by the optimisation. We are interested in whether for a given subject and challenge, optimising the model for another challenge from the same subject improves the model performance compared to the unoptimised model. Let 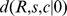 refer to the scaled distance between model prediction and data for signal 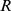, subject 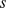 during challenge 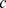, using the unoptimised model and 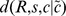 refer to the corresponding distance following optimisation of the model for subject 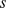, challenge 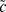. So, for example, 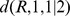 would refer to the predicted distance in 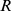 for subject 1, challenge 1, following optimisation for subject 1, challenge 2. Then the percentage
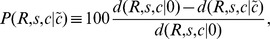
provides a measure of the improvement in prediction of R for subject 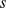 during challenge 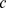 given knowledge of challenge 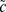. 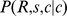 can be regarded as a measure of the success of the optimisation process for a particular signal. 100% represents maximum improvement, that is an exact match between model-prediction and data, post-optimisation, while values less than zero represent a worsening of the prediction following optimisation compared to predictions of the default model. This latter possibility is not unrealistic: it can occur that in attempting to find the best fit to all five quantities, the fit to some of the quantities actually worsens. Hence the importance of appropriate choice of the weights 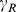. For subject 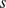 and signal 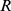 we can compute the quantity:



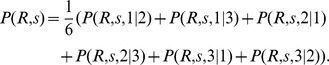


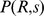
 will be termed a prediction factor. It quantifies the average increase in predictability of signal 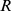 during a challenge on subject 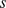 given knowledge of one other challenge for the same subject.

## Results

### Behaviour of the Unoptimised Model

**Figure 4:**
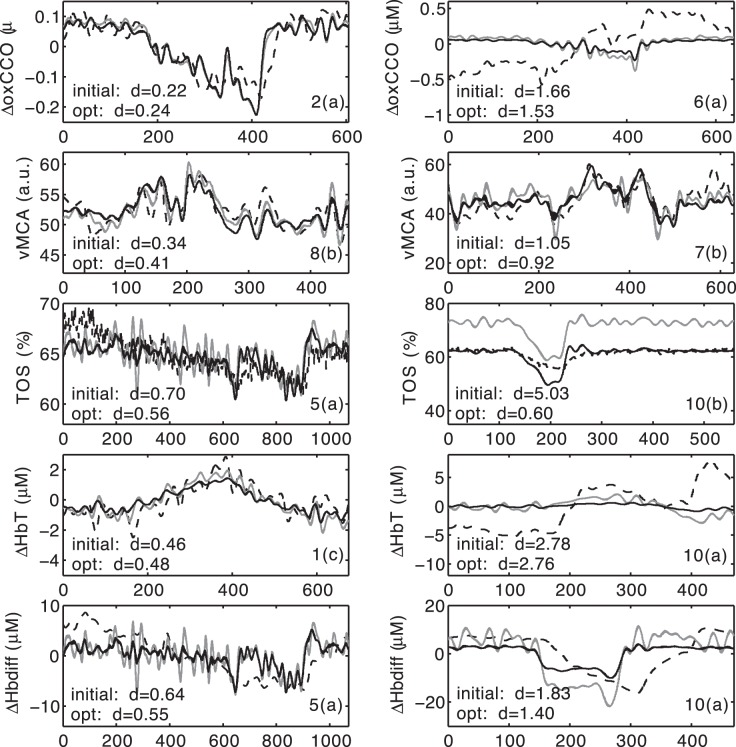
Best and worst performance of the unoptimised model and optimisation results for each signal. Bold lines are the model output post optimisation, grey lines are the unoptimised model output, while the dashed lines are measured data. The best fit prior to optimisation is on the left, while the worst fit is on the right. The weighted distances 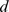 are on the bottom left of each plot, while the subject and challenge are on the bottom right (e.g., “2(a)” means “Subject 2, first challenge”).

Prior to discussing model optimisation and individualisation, it is important to explore briefly the ability of the model *with default parameter values* to reproduce the five signals. This was found to be widely variable, both across subjects and across the different signals with, however, some consistent trends. For illustrative purposes we have chosen the individual challenges corresponding to the best and worst fits pre-optimisation for each of the signals. The results are shown in Fig. 4. The weighted distances from model to data are also shown in each case.

Fig. 4 illustrates that even prior to optimisation the model predictions were sometimes remarkably close to the data. However there was considerable variation in this success, both across subjects, and across signals. For example TOS showed many of the worst initial fits as a consequence of the fact that its baseline varies widely. Subject 10 showed some of the worst fits across a range of signals. Each signal is examined in more detail in the discussion.

### Summary of Results of the Optimisation Process

Figure 4 shows the effect on the model performance of optimisating each signal individually for those signals which showed the best and worst performances prior to optimisation. The signals for which the model had the best performances prior to optimisation had on the whole only small improvement post optimisation (and in some cases show small deterioration in performance). The signals for which the model showed the worst performance prior to optimisation highlight the level of variation seen post optimisation, with some signals showing large improvements (e.g. TOS) and others showing only small improvements 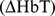. These results are illustrative of the range of results seen, Table 2 shows the average values of 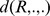 for each of the signals, across all of the challenges. The values in Table 2 can be interpreted in the following way: the optimisation process makes the most substantial difference to the signals TOS and 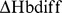. Thus a mismatch between modelled and measured values of TOS and 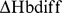 may be at least partly attributable to the choice of model parameters. On the other hand, the process does not considerably improve ΔHbT, vMCA and ΔoxCCO.

**Table 2:** The (weighted) signal-to-data distances for each of the five measured signals, and the sum of these distances, averaged across all thirty challenges for Fit 1.

	TOS	vMCA	ΔoxCCO	ΔHbT	ΔHbdiff	d_tot_(Fit 1)
Unoptimised:	1.87	0.60	0.81	1.11	1.10	5.47
Single-signal:	0.60	0.40	0.71	0.80	0.53	–
Fit 1:	0.70	0.55	0.78	1.02	0.66	3.72
Fit 1(% improvement):	62%	8%	3%	7%	40%	32%

The first row contains these distances for the unoptimised model. The second row shows the results following optimisation to each signal separately. These numbers quantify the maximal ability of the model-class to reproduce the signals individually. The third and fourth rows represent the distances and percentage improvement following collective optimisation of the signals during Fit 1. As we would expect, these distances are consistently higher than those from the single-signal optimisations.

In the case of the CCO signal, optimising this signal individually gives a 12% improvement in fit, while the collective optimisation causes an insignificant change. This should not be interpreted to mean that the model was unsuccessful at predicting CCO: in fact in 14/30 challenges 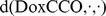 were less than 0.7 prior to optimisation, which corresponds, given the low signal-to-noise ratio of the CCO signal, to a reasonable fit. Rather, altering parameters from the parameter set chosen does not appreciably improve the 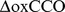 fit on average.

For 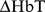 and vMCA, the outcome of the optimisation process across challenges was very variable with maximum improvements of 47% 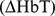 and 40% (vMCA), but also noticeable deterioration in the fits for some subjects. The 28% and 33% improvements following the respective single-signal optimisations, compared to negligable improvements for collective optimisation, suggests that there are difficulties associated with simultaneously optimising the signals. It should be noted that 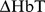 changes were on the whole considerably smaller than 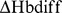 changes during this challenge (see Fig. 4 for example), implying a greater signal-to-noise ratio as both are derived from the same quantities and measured in the same units. We return to these issues in the discussion.

### Improvement in Prediction Following Optimisation

We can term the process of optimising a model to partial data from an individual **model individualisation**. The model individualisation in this case involved optimising the model for one challenge thus, allowing the remaining two challenges for that experiment to be predicted. The success of model individualisation is quantified by the prediction factors 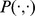. The prediction factors for each signal, and for their weighted sum, are presented for each subject in Table 3, showing that on average there is a considerable improvement in the prediction of two signals, TOS and 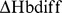, following model individualisation. This is consistent with the results obtained from optimising each challenge individually, implying knowledge of the data during one challenge for an individual improves the ability of the model to predict the behaviour of these signals in subsequent challenges.

**Table 3:** The prediction factors 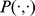 for each subject and each signal following Fit 1.

Subject	TOS	vMCA	ΔoxCCO	ΔHbT	ΔHbdiff	dtot
1	20.3	−4.9	−4.2	2.9	36.2	17.1
2	76.0	−8.4	−11.3	19.9	57.7	48.9
3	−15.5	23.3	2.3	−16.0	22.7	3.0
4	32.2	6.7	−0.1	−8.2	47.0	15.0
5	10.9	11.9	4.9	2.0	7.1	6.5
6	65.1	−14.9	−2.0	12.0	38.6	37.5
7	39.3	6.1	−6.6	11.8	32.4	20.6
8	3.3	3.7	−0.1	3.9	20.1	6.1
9	50.4	−29.8	−0.1	10.8	50.5	29.1
10	81.3	10.3	−2.2	−7.3	28.3	44.0
average	36.3	0.4	−1.1	3.2	34.1	22.8

The table is read as follows: consider the TOS entry for subject 4 (i.e., 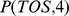) which has value 32.2. This value means that the prediction of the value of TOS for Subject 4 during some challenge was on average 32% improved by model optimisation to data from a different challenge.

### Predicting TOS Change during Hypoxia

Following analysis of the results of Fit 1, a systematic discrepancy between modelled and measured TOS was identified. While optimisation led to successful fitting of baseline TOS, the model, both prior to and post optimisation, consistently overestimated the TOS drop during hypoxia. This occurred despite the fact that arterio-venous volume ratio and haematocrit were free parameters in the optimisation. The single-signal optimisations for TOS were slightly better able to reproduce the observed signal (see Table 2), but the systematic discrepancy remained. Thus it appears that the comparatively low observed drops in TOS during hypoxia were not compatible with the model variation allowed in Fit 1.

**Figure 5:**
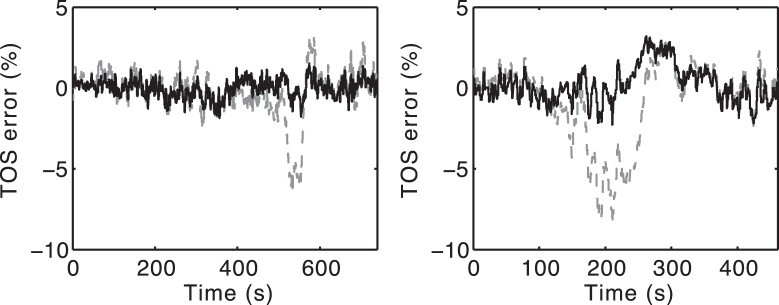
Error in predicted TOS during two challenges for Fit 1 and Fit 2. The TOS error is defined as TOS (model) - TOS (data). TOS error is shown following Fit 1 (dashed line) and Fit 2 (bold line) for two example challenges (Subject 2, challenge 1 and Subject 8, challenge 2). The plots illustrate that optimisation 2 considerably reduced the error in the prediction of TOS by reducing the expected drop in TOS during the hypoxemic challenge.

In order to explore this discrepancy, a new quantity termed 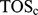, a corrected TOS defined as a weighted average between a fixed default TOS and actual model-predicted TOS, was constructed. One of the original set of seven free parameters 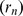 was replaced with the weighting used in the definition of 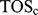, termed 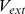, and a second optimisation was carried out. This second set of optimisations is termed **Fit 2**. Table 4 shows the average values and % change of 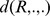 for Fit 2. This second optimisation resulted in considerably better fits to the TOS data, examples of the improvements observed are shown in Fig. 5 where the distance between modelled and measured data is noticeably decreased compared to Fit 1. When comparing the remaining signals with Fit 1, vMCA and 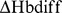 both show improvements in the average results, 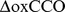 shows no change and 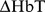 shows that on average there is a slight decrease in the predictive capabilities of the model. As with Fit 1 the results for 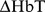 were still extremely variable with a maximum improvement of 35%.

**Table 4:** The (weighted) signal-to-data distances for each of the five measured signals, and the sum of these distances, averaged across all thirty challenges for Fit 2.

	TOS_c_	vMCA	ΔoxCCO	ΔHbT	ΔHbdiff	d_tot_(Fit 2)
Unoptimised:	1.76	0.60	0.81	1.11	1.10	5.36
Fit 2:	0.44	0.50	0.78	1.13	0.63	3.48
Fit 2 (%change):	75%	17%	3%	−2%	43%	35%

The first row contains these distances for the unoptimised model. The second and third rows represent the distances and % change following collective optimisation of the signals during Fit 2.

To highlight the impact on model individualisation Table 5 presents the prediction factors for each signal, and their weighted sum, for Fit 2. The overall values for Fit 2 are slightly better than those for Fit 1, with an average improvement in prediction of 22.8% (Fit 1) and 24.6% (Fit 2). Moreover an improvement in prediction of TOS after Fit 2, compared to Fit 1, occurred for almost all subjects (see Tables 3 and 5), with very marked improvement in some cases.

**Table 5:** The prediction factors 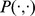 for each subject and each signal following Fit 2.

subject	*TOS_c_*	vMCA	ΔoxCCO	ΔHbT	ΔHbdiff	dtot
1	8.5	−13.7	3.7	4.3	38.9	14.7
2	82.6	−6.2	−13.3	−25.8	62.1	46.2
3	−14.7	23.3	2.2	−20.0	26.6	4.0
4	28.1	21.2	2.2	−10.1	52.2	16.8
5	43.4	7.0	2.8	−9.6	11.6	13.1
6	70.0	7.4	−0.4	−9.2	50.0	38.7
7	50.9	−7.6	−1.9	9.3	23.6	22.9
8	3.6	6.3	−0.2	0.8	25.9	7.6
9	66.4	11.4	−2.1	−0.3	51.3	34.2
10	89.2	20.4	1.3	−10.1	29.0	47.9
average	42.8	7.0	−0.6	−7.1	37.1	24.6

Note that improvements in the prediction of TOS and 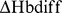 were often accompanied by a slight worsening of the prediction of 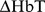.

### Robustness of Parameters Determined from Optimisation

One important question is whether certain clinically relevant parameters, for example the strength of pressure autoregulation, can be determined robustly via the optimisation process from data of the kind presented here. Given some parameter 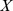, the following questions are relevant:

- How does the average value of 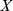 determined by optimisation compare to the default values of 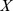 used in the model?
- What is the variation in 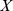 across the 30 challenges?
- What is the variation in 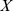 for an individual across the three challenges?

Table 6 summarises the answers to these three questions following Fit 1 and Fit 2 (model default values are given in Table 1). Some parameters showed considerably wider variation across the thirty challenges than others, quantified in the third and sixth columns of Table 6. For example the standard deviation in the optimal values obtained for 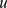 following Fit 1 was 68% of the mean value. A large value like this may reflect different facts:

**Table 6:** Values of the free model parameters following optimisation.

	Fit 1			Fit 2		
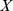	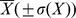	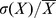	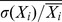	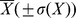	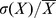	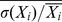
VARn	0.93(±0.39)	0.42	0.27	0.94(±0.34)	0.37	0.16
[Hb]	0.58(±0.17)	0.30	0.15	0.59(±0.15)	0.25	0.10
*r_n_*	0.92(±0.14)	0.15	0.11	–	–	–
*u*	1.09(±0.75)	0.68	0.45	0.87(±0.55)	0.64	0.43
*R_o_*	1.90(±0.99)	0.52	0.34	1.17(±0.67)	0.57	0.40
*R_p_*	1.15(±0.42)	0.36	0.20	1.21(±0.30)	0.25	0.14
*τ_p_*	0.13(±0.13)	0.97	0.48	0.14(±0.10)	0.69	0.56
*V_ext_*	–	–	–	0.42(±0.14)	0.33	0.25

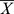
 represents the mean value of parameter 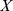 across all thirty challenges. In all cases except 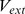, the value is divided by the default parameter value. Thus a value close to 1 implies that the model default is consistent with the data from the study. 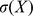 is the population standard deviation of values of 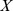 across all thirty challenges. A low value of 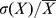 implies that parameter 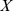 does not show great variation between subjects. 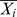 represents the mean value of parameter 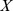 across the three challenges for individual 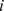 (normalised, except for 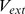). 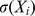 represents the population standard deviation of values of 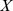 across all three challenges for individual 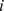. Low values of 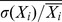 suggest that a parameter can be reliably determined for an individual.

1. It is inherent in the physiological meaning of certain parameters that model behaviour is sensitive to changes in their values, for example they may occur squared or exponentiated in certain model equations;
2. There can be naturally occurring differences in the amount of physiological variability in certain quantities;
3. Some parameters may be hard to estimate from the data-set in this study, and this is reflected in the high variability.

In order to distinguish between possibilities 2 and 3 above, it is important to examine the difference between population standard deviation as estimated from all 30 challenges (third and sixth columns of Table 6) and the average population standard deviation as estimated from each subset of three challenges on an individual (fourth and seventh columns of Table 6). For every parameter and each optimisation, the latter is lower, suggesting picking a model parameter 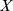 and estimating its value from data for an individual, yields more clustered values than random choice of this parameter from the distribution. This reassures us that partial data from an individual can help us estimate each of the parameters for an individual. It is consistent with the increased predictability of signals from an individual following optimisation using part of that individual’s data.

For the majority of the predicted parameters the average values were reasonably close to their model default values, reflected in the fact that entries in the second and fifth columns of Table 6 are close to 1. However, for certain parameters the predicted averages suggest values which whilst within the expected physiological limits are further from the model defaults. In the case of 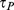, the data consistently suggested values closer to 0.5 second, as opposed to the default model value of 5 seconds. The blood concentration of haemoglobin (haematocrit) [Hb], was also consistently lower than model default of 9 mM and was on average closer to 5 mM. The results also indicated a slightly stronger CBF response to arterial 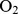 levels. The weighting 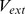 given to default model-predicted TOS in Fit 2 can be seen as a damping factor, a damping factor of 0% would correspond to measured TOS responding as predicted by the model, while 100% would correspond to completely unresponsive TOS. The optimisation process resulted in an average damping factor of 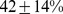, illustrating the consistency of the mismatch between model and data.

### Model Prediction of Unmeasured Quantities: 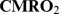


A key goal of the original modelling work was to create a model capable of informing on the behaviour of quantities which are hard to measure in clinical situations. One quantity which is both hard to measure noninvasively and is of clinical importance is 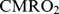. According to the model moderate hypoxia, as carried out here, resulted in a small but not negligible percentage drop in 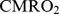. The model predictions, prior to optimisation, were remarkably consistent and were not appreciably altered by the optimisation procedure. All thirty challenges resulted in small percentage drops in 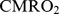, the values of this percentage drop were:

- Unoptimised model: 5.50 ± 1.01 (range: 2.37% to 7.58%)
- Fit 1: 5.92 ± 1.33(range: 2.11% to 9.49%)
- Fit 2: 5.73 ± 1.10 (range: 2.27% to 9.34%)

**Figure 6:**
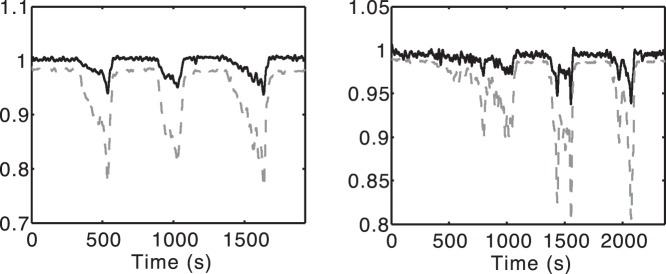
Two examples of measured 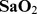 and model-predicted 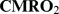 during an experiment. The model predictions are without optimisation. In each case the dashed line is 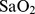 as a ratio, while the bold line is 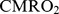 normalised to its initial value. *Left.* Subject 1. This is a fairly typical trace. *Right.* Subject 5. Both 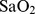 and 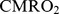 are more variable, but again the model predicts that 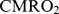 changes follow the trends in 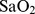.

The maximum change to the value of any particular drop as a result of optimisation was small, with the majority of optimisations having little effect on the model-predicted 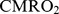 drop. Examples of 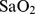 (measured) and 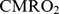 (model predicted) during an experiment are shown in Fig. 6. Discussion of this prediction, and its relationship to the literature, can be found in the concluding section.

## Discussion

We have shown that the BrainSignals model has some success at simultaneously reproducing qualitative and quantitative behaviour of five measurable physiological signals during a hypoxemia challenge. Further, model optimisation can be used to improve model predictions for an individual. In this concluding section, we first provide detail summarising the behaviour of the different signals, as measured and as predicted by the model, and highlighting any systematic discrepancies. We then explore some possible origins of these discrepancies, and the extent to which they could be resolved by altering model assumptions. Finally, the optimisation process itself is examined in order to draw conclusions about model parametrisation.

### Behaviour of the Measured and Modelled Signals

A number of open questions exist in the literature about the reliability, reproducibility, and interpretation of NIRS signals in the context of various physiological challenges (see references in the introduction). Provided some caution is exercised, there is the potential for model-based approaches to inform on these debates. Below, comments on each of the five measured signals are presented and the question of how model-predicted changes in 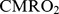 relate to previous observations in the literature, is briefly discussed.

#### ΔoxCCO

This is probably the signal with the lowest signal-to-noise ratio, and also the signal which is hardest to interpret physiologically. In this context, the unoptimised model was relatively successful at reproducing the observed signal. However the optimisation process did not greatly reduce the model-to-data distance, either when carried out on the signal in isolation, or in combination. For certain individuals, the signal prior to detrending shows drifts and/or large fluctuations (in the region of 1 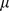
M or more) which are not reflected in the other signals (data not shown), and it is possible that the signal processing carried out here is insufficient to allow robust quantitative analysis when this is the case. Interestingly, it is fairly easy to identify, without reference to the model, experiments where the 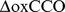 signal appears to be inconsistent with physiological expectations.

#### vMCA

Inspection of the data suggests that this signal is fairly consistent with model predictions both before and after optimisation. While the optimisation process did not cause a large improvement in the match between the modelled and measured data, this can be at least partly attributed to the fact that the model generally successfully predicted changes in vMCA even before optimisation. Unlike previous modelling work [29], [33], the BrainSignals model ignored possible changes in middle cerebral artery diameter during dilation. The data from the present study broadly justifies this choice.

#### TOS

This was the signal to which optimisation made the biggest difference, primarily because baseline TOS varied widely. On the other hand the model, both before and after optimisation, could not account for the comparatively small drops in TOS observed during hypoxia. The heuristic assumption that observed TOS changes were “damped” (Fit 2) led to a considerable improvement in fit. This is discussed further in the following section.

#### ΔHbdiff

This was another signal which even prior to optimisation had a reasonable match between measured and modelled values. It was also the signal to which the optimisation process made the second largest difference. The worst fit shown in Fig. 4 is not consistent behaviour and appears to be more related to the time-course of the 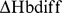 drop during the hypoxemic challenge than its magnitude. As a difference between two measured signals (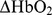 and 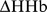) it is possible that some correlated measurement errors cancel, leading to an improved signal-to-noise ratio.

#### ΔHbT

This signal was of fairly small magnitude, consistent with the the relatively small increase in vMCA, and suggesting that vascular dilation caused by hypoxia of this magnitude does not cause large blood volume changes. The relatively large (weighted) model-to-data distance for some subjects may be partly explained by small absolute changes in 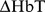. However this may not be the whole story: when optimised singly, model and data could be better matched than when the optimisation included all signals. This suggests that there may be confounding physiological (as opposed to measurement) effects.

#### ΔCMRO_2_

This was not directly measured, but, as mentioned previously, the model was quite consistent in its prediction that drops in 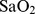 of the order of 15–20% should lead to small but not negligible drops in 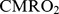. Ref. [37] suggests that while severe hypoxia causes significant decreases in 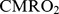, moderate hypoxia in the range considered here does not alter 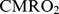. On the other hand, it is worth noting that a drop of a few percent is within the margins of error in the data in [37]. Moreover, the challenges were carried out in anaesthetised dogs, where the average CBF increase observed (150%) during the moderate hypoxia considerably exceeds the average CBF increase inferred from the vMCA data in our study, possibly compensating for the drop in oxygen tension. The data for anaesthetised and awake rats in [38] is similarly inconclusive, suggesting that hypoxia in the range considered here caused significant decreases in 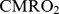 under anaesthetised conditions but not under awake conditions. Again, it is not clear whether changes in 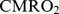 in the 5% range could be reliably estimated by the biophysical BOLD model in question. In sum, it remains an open question whether moderate hypoxia causes a small, but non-negligible, change in 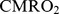.

### Explaining Systematic Discrepancies between Model and Data

The compatibility of a data-set with a model class is represented by the ability of a model to reproduce the data-set following optimisation. When optimisation cannot adequately reproduce a data-set, there may be conceptually distinct explanations:

- Incompleteness of the physiological model class: certain physiological effects are missing from the model, or alternatively, the parameter set chosen for optimisation does not allow sufficient room to obtain the observed behaviours.
- Signal misrepresentation in the physiological model: the physiological interpretation of certain signals is incorrect or incomplete. For example, the two-compartment characterisation of TOS as the weighted sum of arterial and venous haemoglobin saturation, may be too simplistic.
- Signal misinterpretation in the measurement model: the modelling of the measurement process external to the physiological model may be incomplete or flawed, leading, for example, to incorrect translations of raw NIRS data into concentrations or concentration changes.

With these broad principles in mind, we focus in on the most marked and consistent discrepancy between model predictions and data, which concerned the behaviour of TOS during hypoxia. The origin of the discrepancy between model predicted TOS and observed TOS remains unclear, and given the reproducibility of the TOS signal is perhaps the most important discrepancy to explain. Based only on the definition of TOS, it is possible to gain some insight into why model-predictions for TOS drops during hypoxia exceed the observed drops. Indeed, a simple interpretation of TOS as a weighted sum of arterial and venous saturations allows calculation of the approximate changes in TOS which might be observed during hypoxia. Define 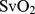 to be mean venous oxygen saturation, and
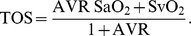
Assume initially 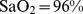 and 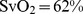 and 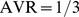, giving 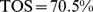. Given an 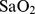 change, by simple conservation of 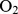, the ratio of initial to final 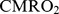 is simply the ratio of initial to final CBF multiplied by the ratio of initial to final arterio-venous saturation difference. For example, assume that a drop in 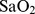 down to 80% occurs, which leads to a 5% drop in 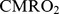, a 10% increase in CBF (as seen approximately in the vMCA data), and a 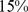 increase in AVR due to arterial dilation. This results in 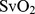 dropping to approximately 48% and TOS to about 57%. This 13–14% predicted drop compares with a 7–8% drop actually observed in the data. An explanation based on physiology is possible, but requires one or more of:

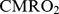
 drops to be greater than predicted;
2. CBF changes to be larger than reflected in the vMCA changes;
3. Arterio-venous volume ratio changes during hypoxia to be considerably larger than predicted.

Each of these possibilities is hard to justify. Further, the damped response of TOS is consistent with preliminary studies on the simultaneous response of TOS and vMCA to changes in inspired 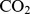 levels [39]. In this case, increased 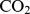 causes significant increases in vMCA, and hence presumably in CBF, which are not visible to the expected degree in the TOS signal.

### Conclusions about Model, Physiology and Pathophysiology

Several of the difficulties inherent in pursuing clinically-directed modelling work have been previously described [40]. Of these, the most pressing involves setting the boundaries of the model class, that is choosing what physiology to include in the model and defining “tolerances” for the values of model parameters. To this end the range of behaviours associated with healthy physiology are examined to ultimately inform on pathophysiology. In this study, we have made progress in these directions, in particular towards finding average values and ranges of variation of model parameters consistent with measured data from healthy volunteers. Progress has also been made towards identifying and explaining areas of discrepancy between model and data.

#### Model Parametrisation

In some cases the optimisations suggest that model default values of certain parameters need to be corrected. For example, the expected delay in the CBF response to blood pressure changes is not observed in NIRS or TCD data, suggesting short-timescale autoregulatory processes, perhaps superimposed on the slower ones. The model optimisations also indicate a lower default haematocrit. Measurements taken from each of the subjects prior to the start of the study gave an average blood concentration of haemoglobin of 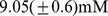 which is in line with the model default of 9.1 mM. The average value post optimisation of 5.27 mM may at least in part be attributed to the differences between venous and cerebral haematocrit. Studies have consistently reported cerebral haematocrit as lower than peripheral venous haematocrit [41], [42] suggesting the need for a change in the model default value. The exact relationship between venous and cerebral haematocrit is not however, straightforward [42] and further analysis is required to determine by what magnitude this parameter should be reduced. Similarly, the optimisations suggest somewhat stronger regulatory response to arterial 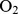 levels than previous model default values. Of course, given the small number of individuals in the study such suggestions about average values need to be treated cautiously. Nevertheless, they provide suggestions for improvements to model parametrisation.

#### Confidence in Model Parameter Values

The values determined by optimisation provide some insight into the potential variability to be expected in model parameters. For example, the results on haematocrit tell us that variations of the order of 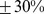 around the default value (Table 6) are consistent with haemoglobin-related NIRS data from this study. On the other hand variability in the predictions for a single subject are 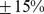, suggesting that some of the 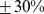 variation can be attributed to genuine inter-subject variability, as opposed to, say, measurement noise. The same general conclusion is true for the other parameters.

#### Information of Clinical Relevance

An important question is what meanings can be ascribed to parameter values obtained for an individual from the optimisation process. For example, even though this study did not explicitly involve blood pressure manipulations, it is instructive to ask what conclusions can be drawn from this data about a subject’s ability to regulate CBF in response to hypoxia and changes in blood pressure. The rows relating to the quantities 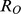 and 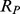 in Table 6 provide an answer to this question. The lower intra-individual variation compared to the total group variation of 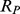 is marked, suggesting in theory, we can distinguish between subjects with a weak or strong CBF response to pressure changes. The results of CBF regulation in response to blood gas changes were less robust. The variation in values for 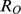 was considerably larger than that in values of 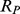. This indicates that extracting reliable estimates of 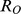 for an individual is a harder task than extracting reliable values of 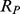. This may also reflect the fact that each rapid fluctuation in blood pressure provides data to estimate 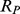, whereas each challenge involves essentially a single drop in 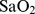, and thus the effective volume of data from which 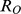 is being estimated is much less. An interesting question is whether inter-subject variability in these quantities may have clinical significance, a question which can only be answered in the context of applying the same methodologies to pathophysiological data.

### Final Comments

The results suggest we can draw conclusions about the methodology which go beyond the BrainSignals model. We have shown that attempting to simultaneously interpret a number of signals with the aid of a physiological model is a challenging task, but with considerable potential reward, particularly in providing clinically relevant information at the bedside to aid patient management. Despite a variety of limitations and difficulties, we can conclude that tackling a large problem (here, optimising seven free parameters using data from five measured signals) is not unfeasibly ambitious. At the very least, via this process, it is possible to increase the predictive capabilities of a model for an individual. Perhaps more importantly, the optimal parameter values may themselves provide clinically useful information. Regarded collectively, exploration of this kind can provide a handle on the range of variability in model parameters consistent with data from healthy volunteers, a crucial precondition for further study in pathophysiological situations.

Several natural next steps exist, building on this work. One question is whether applying the same methodologies to data obtained from other challenges, for example during hypercapnia, or during blood pressure manipulation, gives conclusions consistent with those from this study. Another question is what we discover if the same techniques are applied to pathophysiological data.
